# HPLC-Based Mass Spectrometry Characterizes the Phospholipid Alterations in Ether-Linked Lipid Deficiency Models Following Oxidative Stress

**DOI:** 10.1371/journal.pone.0167229

**Published:** 2016-11-28

**Authors:** Robin Drechsler, Shaw-Wen Chen, Blair C. R. Dancy, Lena Mehrabkhani, Carissa Perez Olsen

**Affiliations:** Division of Basic Sciences, Fred Hutchinson Cancer Research Center, Seattle, Washington, United States of America; Scripps Research Institute, UNITED STATES

## Abstract

Despite the fact that the discovery of ether-linked phospholipids occurred nearly a century ago, many unanswered questions remain concerning these unique lipids. Here, we characterize the ether-linked lipids of the nematode with HPLC-MS/MS and find that more than half of the phosphoethanolamine-containing lipids are ether-linked, a distribution similar to that found in mammalian membranes. To explore the biological role of ether lipids *in vivo*, we target fatty acyl-CoA reductase (*fard-1)*, an essential enzyme in ether lipid synthesis, with two distinct RNAi strategies. First, when *fard-1* RNAi is initiated at the start of development, the treated animals have severely reduced ether lipid abundance, resulting in a shift in the phosphatidylethanolamine lipid population to include more saturated fatty acid chains. Thus, the absence of ether lipids during development drives a significant remodeling of the membrane landscape. A later initiation of *fard-1* RNAi in adulthood results in a dramatic reduction of new ether lipid synthesis as quantified with ^15^N-tracers; however, there is only a slight decrease in total ether lipid abundance with this adult-only *fard-1* RNAi. The two RNAi strategies permit the examination of synthesis and ether lipid abundance to reveal a relationship between the amount of ether lipids and stress survival. We tested whether these species function as sacrificial antioxidants by directly examining the phospholipid population with HPLC-MS/MS after oxidative stress treatment. While there are significant changes in other phospholipids, including polyunsaturated fatty acid-containing species, we did not find any change in ether-linked lipids, suggesting that the role of ether lipids in stress resistance is not through their general consumption as free radical sinks. Our work shows that the nematode will be a useful model for future interrogation of ether lipid biosynthesis and the characterization of phospholipid changes in various stress conditions.

## Introduction

**Fig 1:**
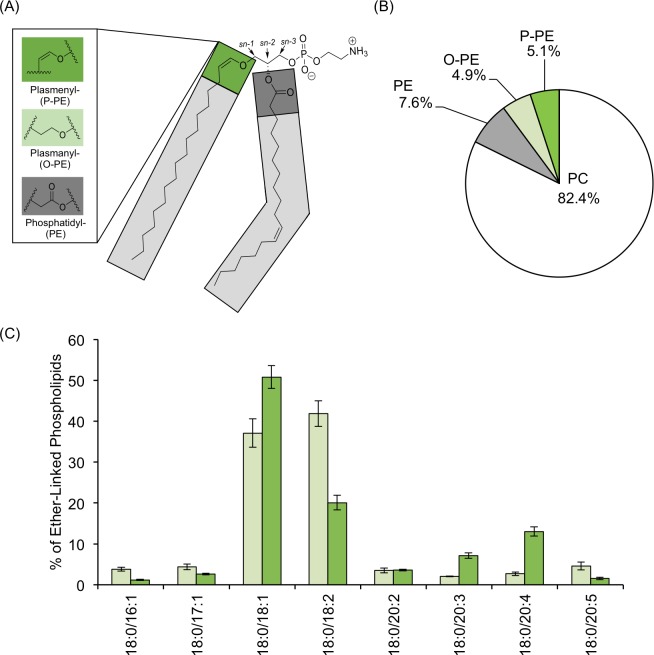
Characterization of Ether-Linked Phospholipids in Adult *C*. *elegans*. (A) The linkage at the *sn-1* position of a phosphoethanolamine lipid can vary, producing: phosphatidylethanolamine (PE), plasmanylethanolamine (O-PE) and plasmenylethanolamine (P-PE). (B) Total lipid content was analyzed by HPLC-MS/MS, and the distribution of major lipids was determined after correcting for ionization efficiency with externally provided standards. The resulting distribution in adult animals is as follows: 82.4 ± 1.4% phosphatidylcholine (PC), 7.6 ± 0.9% PE, 4.9 ± 0.4% O-PE and 5.1 ± 0.6% P-PE. No significant plasmanylcholines (O-PC) or plasmenylcholines (P-PC) were detected. (C) The fatty acid chains (denoted by X:Y, where X represents the number of carbons, and Y is the number of double bonds) linked at each position are reported for both O-PE (light green) and P-PE (dark green). A 0.1% cutoff was used to evaluate major contributors; however, P-PE 18:0/20:2 was included, despite not meeting the minimum criteria in order to have a complementary data set for the 18:0/20:2 O-PE species. Data were generated from 5 independent biological replicates, and SEM is reported. Complete lists of phospholipid abundance can be found in the S1 Dataset.

The composition of an individual membrane lipid dramatically affects its impact on the membrane landscape. The unique features of phospholipids, including their impact on membrane packing and fluidity, are related to the identity of their head groups and the associated fatty acid tails. In a typical phospholipid, two fatty acid tails are attached to the glycerol backbone via two ester linkages at the *sn-1* and *sn-2* positions (Fig 1A). A feature that expands the diversity of phospholipids is the method by which the fatty acids are connected to the glycerol backbone. In the case of ether lipids, an alkenyl-ether group (plasmenyl (P)) replaces the ester bond at the *sn-1* position. These ether-linked lipids, also called plasmalogens, can be found in a wide range of eukaryotic membranes and comprise nearly 20% of the human phospholipidome [1]. The immediate precursors of the plasmalogen population contain an alkyl-ether bond (plasmanyl (O)) and are detected in significant quantities in the phospholipidome as well [2]. The presence of these P- and O- bonds affects the biophysical properties of the entire phospholipid moiety, specifically making assemblies of these lipids less fluid than ester-bonded counterparts, which may help to modulate responses to external stimuli including changes in temperature [3].

The defining feature of ether lipids is the ether linkage at the *sn-1* position, which not only dictates the properties of the molecules but also is one of the reasons they have been difficult to study, as the alkenyl-ether form is acid labile. Phosphoethanolamine lipids with an alkenyl-ether bond are lost with many standard lipid protocols. Plasmalogens can be quantified indirectly through the measurement of dimethylacetals; however, these derivatives are unstable in some GC-MS conditions [4]. For their direct quantification, ether lipids need to be purified before processing or analyzed intact, which is increasingly feasible with improvements in mass spectrometry technologies. Recently, we have used HPLC-MS/MS methods to map the major phospholipids of the nematode including ether-linked species. Moreover, we described the use of ^15^N-tracers to quantify intact phospholipid dynamics and found that ether lipids have different dynamics than other phosphoethanolamine-containing lipids [5].

As well as influencing membrane fluidity and permeability, the ether-linkage may also be involved in other cellular processes including signaling and antioxidant scavenging. Of course, these roles of ether lipids in the membrane are not mutually exclusive, particularly in how these lipids may impact response to changing conditions such as elevated oxidative stress or temperature shifts. One of the most established models for ether lipid function describes them as sacrificial antioxidants that limit the impact of reactive oxygen species on the membrane [6]. The polyunsaturated fatty acids (PUFAs) of the membrane are particularly sensitive to reactive oxygen species, and, when oxidized, not only is the targeted lipid destroyed, but the resulting lipid peroxides can themselves initiate a cascade of damage that propagates to other membrane lipids and cellular macromolecules [7, 8]. The vinyl-ether-linkage of plasmalogens can serve to trap reactive oxygen species, and it has been demonstrated that singlet oxygen interactions with ether lipids occur faster than with other lipids *in vitro* [9]. Unlike other phospholipids which form reactive peroxides when damaged, the ether lipids may produce less persistent oxidative byproducts including free aldehydes and hydroperoxides [10]. Additionally, the sacrificial antioxidant hypothesis is supported by studies demonstrating that cells lacking ether lipids are sensitive to oxidative stress [1, 2].

Despite the lack of a full understanding of the biological activity of ether lipids, it is clear that the absence of ether lipid synthesis directly results in diseases including Rhizomelic Chondrodysplasia Punctata (RCDP) and other Peroxisomal Biogenesis Disorders (PBDs). Affected individuals clinically present with severe developmental delays, a wide-range of health problems, and early death [11, 12]. Interestingly, in addition to a specific role in PBDs, the abundance of ether-linked lipids is altered in Alzheimer’s disease, schizophrenia, obesity, and cancers, suggesting that they are not only vital for development but that they also may play an important role in adult membranes [13–16]. Therefore, it is essential to increase our understanding of both the regulation and biological functions of ether-linked lipids in order to aid in the improved treatment and prevention of both ether lipid-deficiency disorders and other diseases in which ether lipid perturbation has been correlated.

A recent study isolated *Caenorhabditis elegans (C*. *elegans)* mutant strains containing loss of function mutations for the first three genes in the ether lipid biosynthesis pathway, *fard-1* (fatty acyl-CoA reductase), *acl-7* (glyceronephosphate *O*-acyltransferase), and *ads-1* (alkylglycerone phosphate synthetase). These genes have mammalian homologs, *FAR1*, *GNPAT* and *AGPS* respectively, which are required for ether lipid production and result in stress-sensitive cells when depleted [1, 2]. Analysis of these ether lipid synthesis *C*. *elegans* mutants revealed that the animals are deficient in ether lipids. Additionally, these mutants have gross phenotypes including shortened lifespan and sensitivity to oxidative stress [4]. Here, we employed HPLC-MS/MS in *C*. *elegans* to map the abundance and distribution of ether lipids in adult nematodes. Our study corroborates many of the key findings from Shi *et al* and has allowed us to further the knowledge of ether lipids in the nematode [4]. In particular, we focus on the biochemical quantification of individual ether lipids as well as the other major membrane phospholipids that are perturbed in their absence. Moreover, we have implemented multiple RNAi strategies to selectively target the ether lipid-biosynthesis genes at multiple stages of life. The use of RNAi at different developmental stages has enabled us to further probe the role of ether lipids and their synthesis in the stress response of the nematode. We have created an extensive map of phospholipid remodeling that results from oxidative stress that will allow for the evaluation of unanswered questions regarding the regulation and potential antioxidant properties of this class of phospholipids.

## Results

### Ether-Linked Species are Major Components of the Nematode Phospholipidome

As *C*. *elegans* has recently been introduced as a model for ether lipid deficiency, we sought to determine how the distribution of ether lipids in the nematode compares with humans. In other species, the majority of ether-linkages are found within phosphoethanolamine-containing lipids where the fatty acid bonded at the *sn-1* position can contain an acyl moiety (phosphatidylethanolamine, PE), an *O-*alkyl ether bond (plasmanylethanolamines, O-PE), or an *O-*alkenyl ether bond (plasmenylethanolamines, P-PE) (Fig 1A). Both O-PE and P-PE populations have been identified in *C*. *elegans;* however, their abundance is not yet defined [4, 5]. Many aspects of lipid metabolism including the distribution of lipid classes can change depending on the developmental stage, the reproductive status and the age of the animal. Here, we established day 3 of adulthood as our primary collection stage as these animals have completed development and reproduction (see S1 Fig for brood analysis). Therefore, we exclusively examined the adult phospholipidome. Furthermore, we used an established temperature-sensitive sterile strain, *fer-15;fem-1*, to eliminate the presence of progeny that can contaminate the lipid profiles [5].

Because phosphocholine- and phosphoethanolamine-containing lipids are by far the most abundant phospholipids in the nematode as well as in humans, we considered these classes specifically and identified all the species within by their exact mass, retention time and predicted isotope distribution [5, 17]. In order to ensure equal recovery of lipids with phosphocholine- and phosphoethanolamine headgroups, a panel of external standards was implemented to correct for differences in ionization efficiency in the mass spectrometer (see Materials and Methods). In day 3 adults, phosphatidylcholines are the most abundant phospholipid class in the nematode making up more than up 82.4±1.4% of the major phospholipids; however, we did not detect any significant ether-linked lipids, namely plasmanylcholines or plasmenylcholines, within the phosphocholine-containing lipids (Fig 1B). These species were also not found in significant amounts by a distinct mass spectrometry analysis completed by Shi et al [4]. The absence of ether-linked PC represents a difference in the nematode phospholipidome as humans contain significant amounts of ether-linked PC, particularly in cardiac tissue [2]. Among the PE population, the typical di-acyl lipids represent the majority of the population; however, there are significant amounts of O-PE and P-PE, representing 22.7 ± 1.4% and 30.3 ± 0.9% respectively of the phosphoethanolamine-containing lipids (see S1 Dataset). In considering these major classes, the contribution of ether-linked lipids represents approximately 10% of the major phospholipids in the adult nematode, making these ether-linked species major contributors to the overall phospholipid landscape (Fig 1B).

### Characterization of the Individual Ether-Linked Lipid Species

We further defined the ether-linked population by considering the species of fatty acid present at each position of the glycerol backbone. In *C*. *elegans*, the fatty alcohol attached at the *sn-1* position is a saturated C18 species in nearly all ether-linked molecules as we have previously reported [5] (Fig 1C, see S1 Dataset for complete phospholipid profile). In contrast, the *sn-2* position can incorporate a number of different fatty acid species, with the majority of molecules containing a C18 fatty acid with one (37.1 ± 3.5% for O-PE and 50.8 ± 2.8% for P-PE) or two double bonds (41.9 ± 3.2% for O-PE and 20.1 ± 1.8% for P-PE) (Fig 1C). There are substantial amounts of C20 polyunsaturated chains in the ether lipid pools of adult nematodes, particularly in the P-PE lipids, accounting for 25.3 ± 2.3% of that population (Fig 1C). There are subtle differences in the distribution of lipids reported by Shi et al; however, both analyses show 18:0/18:1 and 18:0/18:2 as the primary fatty acid combinations in both O-PE and P-PE pools [4]. Furthermore, the numbers cannot be directly compared due to the harvest of different stage animals and the implementation of distinct methodologies. Our profiling corroborates the major features of the ether-linked lipids and further establishes the nematode as a model for the study of ether lipids [4].

### Ester-Linked Phospholipid Populations are Altered by Complete Plasmalogen Deficiency

**Fig 2:**
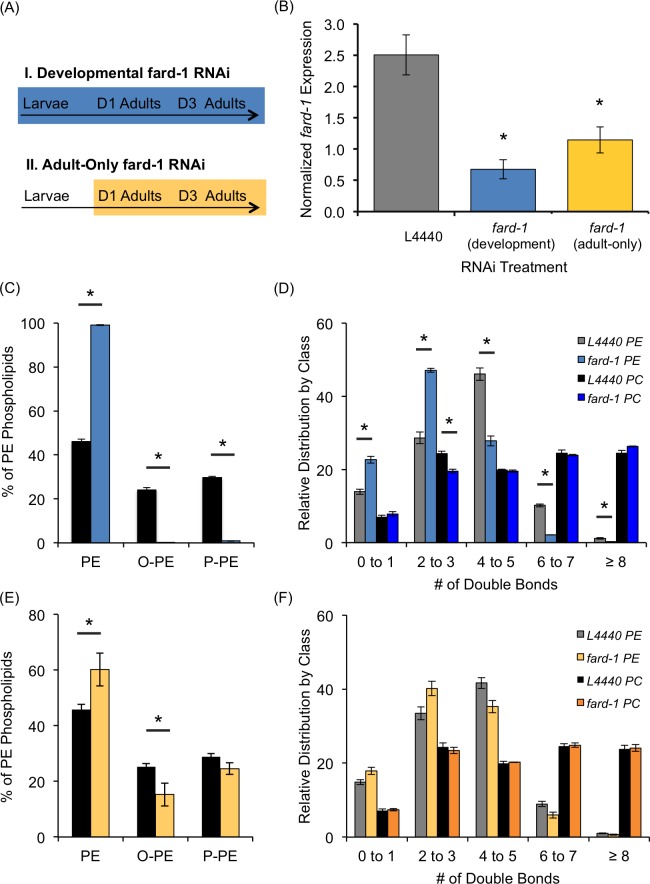
*fard-1* RNAi Treatments Results in Compromised Ether-Linked Lipid Abundance. (A) Developmental RNAi-treatment was initiated at the L1 stage and continued until animals were harvested for analysis at day 3 of adulthood, resulting in 4 days of RNAi feeding at 25°C (blue). The second RNAi-treatment described is an adult-only *fard-1* RNAi strategy (yellow) where RNAi is started after the completion of development at day 1 of adulthood (44 hours post-hatch). Animals were harvested at day 3 for both treatments. (B) qRT-PCR of *fard-1* expression is reduced to 0.34 ± 0.18 and 0.40 ± 0.17 of wild-type levels with developmental RNAi and adult-only RNAi treatment, respectively. The *cdc-42* housekeeping gene was used for normalization. (C) When *fard-1* RNAi is initiated at the L1 stage and continued throughout development until day 3 of adulthood (blue), there is a significant reduction in the relative abundance of both O-PE and P-PE compared to control L4440 RNAi (black). (D) The overall composition of PE was compared in control RNAi- (grey) and *fard-1* RNAi-treated animals (light blue) after binning the lipids by the overall degree of unsaturation found in the associated fatty acids. Similarly, PC species were measured in RNAi controls (black) and *fard-1* RNAi-treated animals (dark blue). (E) *fard-1* RNAi initiated in adult animals at day 1 and maintained until day 3 of adulthood (yellow) resulted in a significant decrease in O-PE as quantified by HPLC-MS/MS. There is a small but not significant decrease in the P-PE population (p-Value of 0.11). (F) The impact of adult-only *fard-1* RNAi treatment on the PE and PC populations showed no significant changes in the degree of unsaturation in PE (yellow) or PC (orange) compared to L4440 RNAi shown in grey for PE and black for PC. Data was generated from at least 3 independent biological replicates with SEM shown. See S1 and S2 Datasets for complete list of phospholipid species. *p<0.05 was determined by unpaired t-tests using Holm-Sidak corrections for multiple comparisons.

The presence of an alkyl or alkenyl bond in a phospholipid changes the properties of that molecule, specifically allowing tighter packing and reducing fluidity [2]. Isolation of loss-of-function mutations in ether lipid synthesis genes in *C*. *elegans* revealed an increase in the amount of saturated fatty acids in all lipid classes which were proposed to compensate for the increased fluidity predicted by ether lipid loss [4]. We sought to further examine the perturbations of the major phospholipids upon ether lipid deficiency using an RNAi model in order to examine the immediate effects of short-term ether lipid deficiency versus the long-term, multigenerational perturbation of the mutant animals. RNAi against *fard-1*, the proposed rate-limiting gene in ether lipid synthesis, was previously found to result in significant changes in ether lipid indicators including an accumulation of 18:0 fatty acid and a depletion of 18:0 dimethylacetal [4]. Here, we directly measured the ether lipids by HPLC-MS/MS and present a complete characterization of the membrane landscape when L1 stage animals were fed *fard-1* RNAi until day 3 of adulthood (Fig 2A). In this developmental *fard-1* RNAi model, we saw a dramatic depletion of ether-linked lipids with over 95% reduction in the abundance of both O-PE and P-PE classes (Fig 2C).

We also examined the defined lipid alterations, namely altered PE levels and increased saturated fatty acid content, in our developmental *fard-1* model. Indeed, the *fard-1* RNAi treatment drove a similar increase in the relative abundance of PE lipids (Fig 2C) similar to what has been observed in fibroblasts derived from RCDP patients and in *fard-1* loss-of-function mutants [4, 18]. Additionally, we observe an accumulation of saturated C18 fatty acids in the total phospholipid compartment and in the neutral lipid population by gas chromatography-mass spectrometry (GC-MS) (see S2 Fig). The accumulation of saturated fatty acids in the neutral lipids is greater than in the phospholipid fraction indicating that the RNAi perturbation of ether lipid synthesis results in an impact on the overall fatty acid saturation within the nematode. The increase in saturated C18 may be a passive consequence of reduced ether lipid production as excess C18 chains would be available in the absence of ether lipid synthesis. Alternatively, it may represent an active remodeling of the nematode’s fatty acid profile to adapt to ether lipid absence, which would predict additional changes in the distribution of phospholipids.

To better understand this compensatory response, we examined the individual phospholipid species by direct sampling with HPLC-MS/MS. In doing so, we focused on the intact phospholipid molecules which allows for the determination of the overall amount of saturation within the lipid and not only the distribution of the chains throughout the class, which would be representative of data obtained from GC-MS. There are significant changes in 11 of the 21 major PE species (see S1 Dataset). To understand the impact on the saturation of the membrane, we binned the phospholipids by the number of double bonds contained within both of their fatty acid tails and found a major shift in the degree of saturation within the PE population (Fig 2D). Specifically, in L4440 control RNAi-treated animals, the largest proportion of PE lipids contain a total of 4 to 5 double bonds; however, in *fard-1-*treated nematodes, PE species containing 2 to 3 double bonds were the largest contributors to the pool (Fig 2D). Species with more than 4 double bonds are all significantly decreased, suggesting a remodeling of the population towards a less fluid state (Fig 2D). Interestingly, the alterations in PE parameters cannot be accounted for solely by the increase in saturated C18 as PE molecules such as 38:6 and 38:7 are significantly altered in the RNAi-treated animals and do not include C18:0 fatty acid tails (see S1 Dataset). The remodeling of the PE population, therefore, is driven by increased saturated fatty acid as well as by additional changes in the distribution of phospholipid species. Ultimately, these altered parameters may stabilize the membrane upon ether lipid loss.

In contrast, although there are minor changes in individual PC species, the overall characteristics of the PC population are relatively unchanged with *fard-1* RNAi treatment (Fig 2D and S1 Dataset). This lack of PC remodeling is significant as PCs make up a far greater proportion of the membrane landscape (>80% PC versus <20% PE; Fig 1B). Taken together, the remodeling of the PE pool may ultimately reduce the fluidity of the membrane; however, it also must be considered that the PE remodeling may also contribute to local membrane structures and/or signaling pathways. In summary, HPLC-MS/MS demonstrates that although the amount of saturated fatty acid is increased in multiple lipid classes, there is more of an impact on the level of saturation in the PE class than in the PC populations, indicating that PE pools are more affected by ether lipid deficiency.

### Targeting Ether Lipid Synthesis Specifically in Adult Animals

A key advantage of an RNAi-based model for ether lipid depletion is that the knockdown can be initiated at specific times during the lifespan of the nematode (Fig 2A). We, thus, can consider for the first time the specific role of ether lipid synthesis in the adult after the formation of membranes during development is complete. To do this, nematodes were grown on control (L4440 empty vector) bacteria until day 1 of adulthood, and, at this point, the animals were moved to *fard-1* RNAi (Fig 2A). The distribution of ether lipids from animals treated with adult-only *fard-1* RNAi were measured with HPLC-MS/MS, revealing a shift in the distribution of ether-linkages (Fig 2E). Specifically, the adult-only *fard-1-*treated animals have a 39.3% reduction in O-PE and 17.3% reduction in P-PE. The reduction in P-PE abundance was not statistically significant; however, this may be due to the relatively short duration of RNAi treatment (Fig 2E, see full list in S2 Dataset). Longer adult-only RNAi feeding did result in further perturbation of the ether lipid population as indicated by increased C18:0 abundance; however, these animals are significantly older (day 8 of adulthood) making direct comparisons of lipid profiles problematic (see S3 Fig).

As with the developmental *fard-1* RNAi, we examined the impact that adult-only *fard-1* depletion had on the PE lipids. Adult-only *fard-1* RNAi resulted in more limited alterations in the lipids with significant changes in only 4 PE species compared to altered abundance of 11 PE species after developmental RNAi (see S2 Dataset). Moreover, the changes in individual species had a minimal impact on the overall saturation found in the lipid classes, and, in fact, there were no statistically significant changes in the distribution of double bonds within either PC or PE pools (Fig 2F). Despite this, there are trends within each population that mirror those seen with the remodeling of PE after developmental RNAi treatment. For instance, there is an increase in phospholipids with 2 to 3 double bonds and a decrease in species with 4 to 5 bonds (Fig 2F). The lack of significant remodeling may indicate an inability for the appropriate changes to be orchestrated in the adult animal.

Because the O-PE and P-PE abundances were less impacted after adult only *fard-1* RNAi, we confirmed the efficacy of our RNAi strategy by qRT-PCR. The expression of *fard-1* is decreased by nearly 60% in the adult-only RNAi protocol which is nearly the same reduction as the 65% observed during the developmental RNAi treatment (Fig 2B). The difference in RNAi efficacy can be explained by less efficient RNAi during adulthood in *C*. *elegans*; however, another consideration is the length of the exposure to RNAi. The developmental *fard-1* RNAi-treatment results in the nematodes being fed RNAi for 4 days with the adult-only treatment resulting in only 2 days of exposure (Fig 2A). This setup was established in order to compare animals of the same age, as many parameters of lipid metabolism vary depending on the age of the animal. The duration of RNAi exposure should be considered by comparing distinct lengths of RNAi treatment with residual expression and ether lipid abundance in future studies.

**Fig 3:**
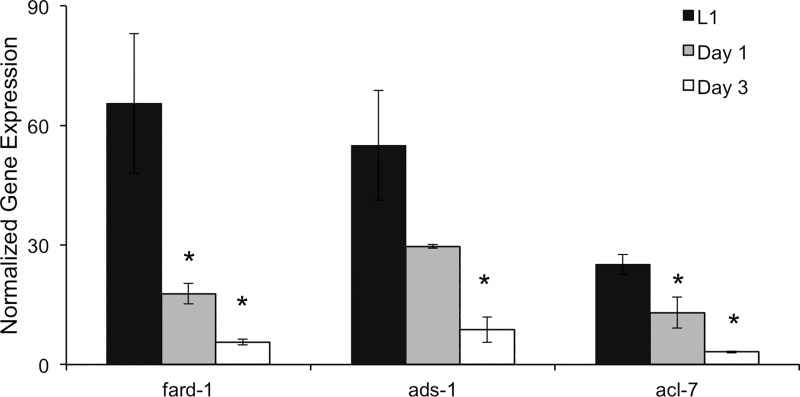
Expression of Ether-Lipid Synthesis Genes is Greatest in Larvae. In wild-type nematodes, the expression of *fard-1* is significantly lower in day 1 (grey) adults and day 3 (white) adults when compared to L1 animals (black). A separate analysis was performed to assay the expression of two additional ether lipid biosynthetic genes, *acl-7* and *ads-1*. The statistical significance was determined by one-way ANOVA with significant differences (p<0.05) being indicated by *. Data is a result of at least 3 biological replicates with SEM shown.

To ascertain how ether lipid synthesis genes are expressed throughout the wild-type animals lifecycle, we measured the expression of *fard-1* along with *acl-7* and *ads-1*, additional ether lipid synthesis genes, in L1 stage animals versus adults. The expression of all the ether lipid synthesis genes was greatest in L1 animals and decreased significantly in day 3 adults. In fact, *fard-1* expression was at least 13-fold higher in the L1 animals (Fig 3). The lower expression in adults argues that the less dramatic phenotypes after adult-only RNAi are a result of a reduced capacity to synthesize ether lipids in the adult stage and not exclusively because of the decreased RNAi efficiency. The trajectory of ether lipid synthesis gene expression highlights the importance of ether lipid production during development and helps to explain the more extreme phenotypes observed in developmental RNAi-treated populations.

### Quantification of Ether Lipid Synthesis in Adult Nematodes

**Fig 4:**
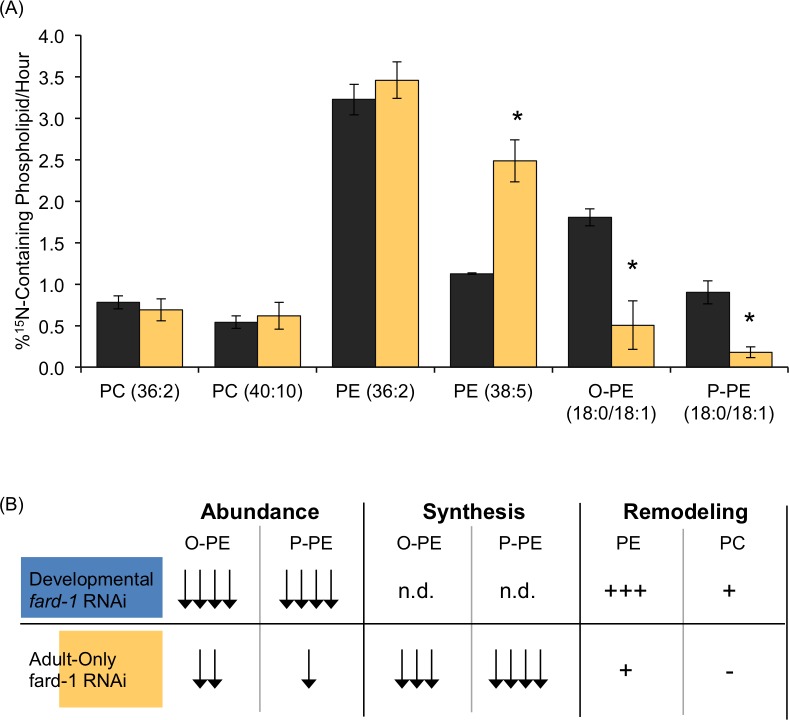
Adult-Only *fard-1* RNAi Treatment Causes Reduced Ether Lipid Production. (A) ^15^N-incorporation assays allowed for the measurement of new phospholipid headgroup incorporation. Phospholipid species are identified here by the summated molecular species that comprise both fatty acid tails. For example O-PE (36:1) represents a plasmanylethanolamine with 36 carbons and one double bond in the associated fatty acids. In *fard-1* RNAi-treated adults (yellow), there is depletion in newly synthesized O-PE (36:1) and P-PE (36:1), but not PC (36:2) or PE (36:2) as compared to control animals (black). Data was generated from at least 4 independent biological replicates with SEM shown. *p<0.05 was determined by unpaired t-tests using Holm-Sidak corrections for multiple comparisons. (B) Here we present a summary of the lipid biochemical data from developmental and adult-only *fard-1* RNAi treatments. Developmental *fard-1* RNAi results in stronger phenotypes across all categories including synthesis where there were not sufficient O-PE and P-PE lipids for analysis.

To understand the role of ether lipid synthesis in adults, we examined the production of new ether lipids with a ^15^N-labeling strategy. The ^15^N-label is incorporated in PC and PE headgroups which both contain a single nitrogen atom, and the accumulation of ^15^N can thus be used to measure newly formed lipids [5]. Indeed, during adulthood, ^15^N accumulates at distinct rates within all of the measured phosphoethanolamine-containing lipids. Ether lipids with a combination of 18:0 and 18:1 fatty acid tails were used in this comparison as they are highly abundant in both alkyl and alkenyl forms, and these species met the standards for analysis with ^15^N-incorporation as previously discussed [5]. We found higher replacement of O-PE (18:0/18:1) than of P-PE (18:0/18:1) in adults (32.5±3.2% versus 16.2±4.4%) (Fig 4A). The faster replenishment of the O-PE compared to P-PE population is somewhat surprising as there are no defined roles for O-PE other than as a precursor for P-PE synthesis. Therefore, the conversion of O-PE to P-PE may represent a regulated and rate-limiting step of ether lipid production, and thus, the precursor alkyl population may accumulate in significant quantities to rejuvenate P-PE pools when needed. This model is supported by the specific decrease in O-PE pools that is observed with adult-only *fard-1* RNAi treatment (Fig 2E). The production of both O-PE and P-PE species occurs at a lower rate compared to PE (36:2) as well as other PE populations previously measured [5]. A direct extension of this analysis would be to measure *de novo* production of ether-linked lipids in larval stages of the nematode; however, the rapid growth of the larval animals makes it difficult to distinguish new membrane formation from maintenance. The expression of ether-linked lipid synthesis genes in the early animal would suggest highly abundant production during development (Fig 3).

Adult-only *fard-1* RNAi treatment resulted in a significant reduction in the amount of ^15^N found in the both O-PE and P-PE classes, indicating that the synthesis of new ether lipid species was significantly compromised by this adult-only RNAi strategy as predicted (Fig 4A). The altered production was specific to ether-linked lipids, as the synthesis of representative species from PC and PE were not compromised (Fig 4A). In fact, the production of PE (38:5) was significantly higher in the *fard-1* RNAi-treated populations consistent with remodeling of the PE populations upon ether lipid synthesis deficiency (Fig 4A). The abundance of PE (38:5) is elevated in adult-only *fard-1-*treated animals; however, the increase is not statistically significant, and the increased ^15^N-incorporation may reflect faster turnover of this population (S2 Dataset). In summary, the adult-only RNAi results in dramatic reductions in O-PE synthesis (73% decreased) and P-PE synthesis (80% decreased). Interestingly, the overall abundance of P-PE remains largely intact despite the reduced capacity to make these lipids. It is possible that the O-PE population is converted to P-PE as a mechanism to preserve P-PE pools, and longer ether lipid deficiency is needed to observe a more dramatic reduction in P-PE. Regardless, adult-only RNAi of *fard-1* is a viable model to probe the function of new ether lipid synthesis in adult membranes.

The RNAi treatments described here have distinct impacts on ether lipid metabolism that will be informative for future studies interrogating the function of ether-linked lipids in disease models. We summarize the biochemical perturbations observed with each RNAi treatment including the abundance of ether-linked lipids, their production and the impact of ether lipid deficiency on other lipid pools (Fig 4B). This comprehensive analysis allowed for the identification of key trends from a complex dataset and found, for example, that the PE remodeling was much more extensive after developmental RNAi treatment. Along with exploring the impact of ether lipid synthesis at different life stages, these models will also be useful in distinguishing the consequences of reduced ether lipid abundance in comparison to compromised synthesis. For instance, ether lipid deficiency results in sensitivity to oxidative stress; however, it is unclear whether ether lipid abundance or synthesis is the primary driver of this impact. The adult-only model can be useful in dissecting these parameters.

### PE Lipids are Impacted by Paraquat Exposure

**Fig 5:**
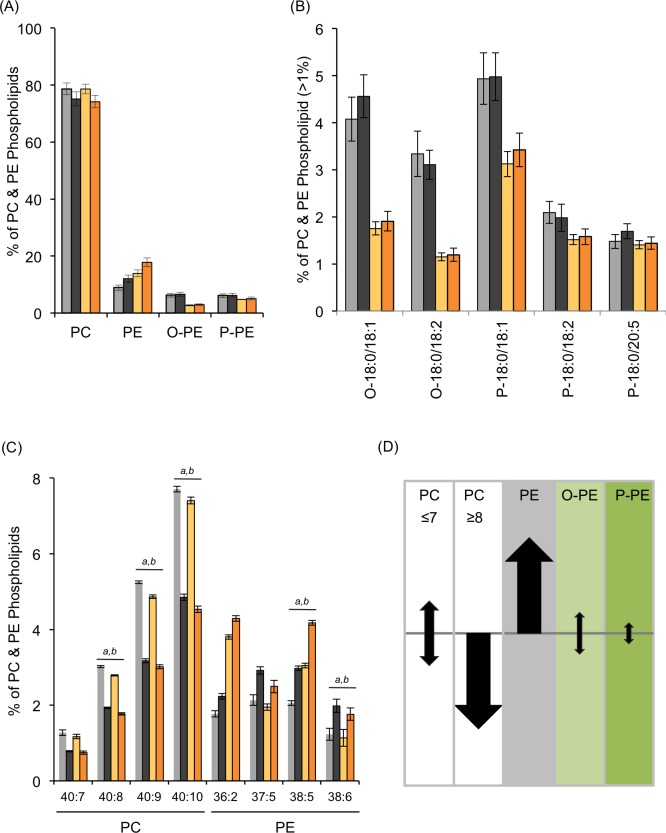
Elevated Oxidative Stress does not Impact Ether Lipid Abundance. The abundance (A) and distribution (B) of O-PE and P-PE within the major phospholipids in L4440-fed animals before (grey) and after (black) 100mM PQ treatment is shown. The same comparisons were done in adult-only *fard-1* treated animals before (yellow) and after (orange) stress treatment. (C) All PC and PE species with significant changes are represented, and the full list is available in the S3 Dataset. Biochemical analysis was done on at least 12 biological replicates with SEM shown. For simplicity, only changes meeting a statistical significance of p<0.001 are denoted here by ^a^ for L4440 RNAi and ^b^ for *fard-1* RNAi treatment, and the complete statistical analysis is found in the S3 Dataset. (D) Here, we summarize the changes in the membrane phospholipids after paraquat treatment. The model presented here was generated by considering the major phospholipid species (≥ 2%), thus removing species that may have exhibited significant changes after PQ treatment but compose a very small fraction of the total phospholipids. The PC pool was divided into a group containing ≤7 double bonds and one with ≥8 double bonds as these populations behaved in distinct manners.

Ether lipids have been implicated in the response to oxidative stress in both mammals and nematodes; therefore, we considered the impact of stressed conditions on the ether lipids using HPLC-MS/MS analysis [2, 4]. To do so, we examined whether the ether lipid populations are directly impacted by exposure to 100mM paraquat (PQ) in wild-type animals. PQ produces elevated levels of oxidative stress, and the sacrificial antioxidant model of ether lipid function predicts that increased oxidative species would consume oxidant-labile molecules and specifically ether lipids. However, HPLC-MS/MS analysis demonstrated that there are no changes in the overall abundance of O-PE or P-PE populations after stress exposure (Fig 5A). We hypothesized that the relatively short exposure of PQ might not result in a detectable depletion of ether lipids; therefore, we also measured the phospholipids in wild-type animals exposed to a longer period of stress. Because PQ is ultimately toxic to the nematode, the 4-day PQ exposure was completed with a reduced concentration of PQ (25mM), and again there were no significant changes in O-PE and P-PE pools (see S4 Fig).

We hypothesized that new ether lipid synthesis could compensate for the consumption of ether lipids by reactive oxygen species (ROS) through the upregulation of *de novo* ether lipid production. As the adult-only *fard-1* RNAi treatment dramatically compromised ether lipid synthesis, this treatment would severely limit the ability to make new ether lipids that could replace those consumed by ROS. In these adult-only *fard-1* treated animals, there was reduced ether lipid abundance as described (Fig 2C); however, there was not any additional reduction due to the PQ exposure (Fig 5A). Therefore, it is clear that increased *de novo* synthesis of ether lipid pools is not responsible for the maintenance of ether lipid levels in wild-type animals exposed to stress. The stable abundance of ether lipids, even in the absence of new production, suggests that their primary role may not necessarily be as global antioxidants in the nematode.

### Specific Phospholipids are Consumed Following Paraquat Treatment

Not only did we quantify shifts in the relative abundance in phospholipid classes with PQ treatment, but we also monitored for changes in the major phospholipid species in wild-type adults. There are no significant changes for any individual O-PE or P-PE species after PQ exposure (Fig 5B, see S3 Dataset for complete list). However, there are a number of significant changes in individual PC and PE species following oxidative stress treatment (Fig 5C). Most notably, we see a significant reduction of PC molecules that contain more than 7 double bonds, specifically PC 40:8, PC 40:9 and PC 40:10 (Fig 5C). This group is comprised of lipids that contain two C20 PUFAs, which are particularly sensitive to oxidative stress, and their depletion supports the efficiency of the PQ treatment implemented here. In the major PE lipids, there are significant increases in PE 38:5 and PE 38:6 populations after treatment (Fig 5C). Overall, the changes in the phospholipid landscape suggest that the primary effect of PQ exposure is a depletion of PC species with multiple PUFA tails and not the oxidant-mediated reduction of ether lipid pools.

We further probed whether compromised ether lipid synthesis impacted the properties of the other major phospholipids and their response to PQ exposure. This analysis was done in adult-only *fard-1* RNAi-treated animals where the starting phospholipid composition is much more similar to wild-type adults. In these *fard-1* animals, there were significant differences in the major PC and PE species; however, the altered species are the same as observed with control RNAi suggesting that the changes are independent of the ether lipid synthesis pathway (Fig 5C). Any subtle changes in the O-PE or P-PE populations would be magnified in *fard-1* treated animals; however, the ether lipids were not significantly different than the controls after PQ treatment (Fig 5B). Our direct analysis of the biochemical changes following PQ treatment suggests that the primary effects of PQ stress are a decrease of PC species with C20 chains and a general increase in PE species, and both of these effects appear to be independent of ether lipid synthesis (Fig 5D).

### Adult-Only *fard-1* RNAi Treatment Results in Temperature Sensitivity

**Fig 6:**
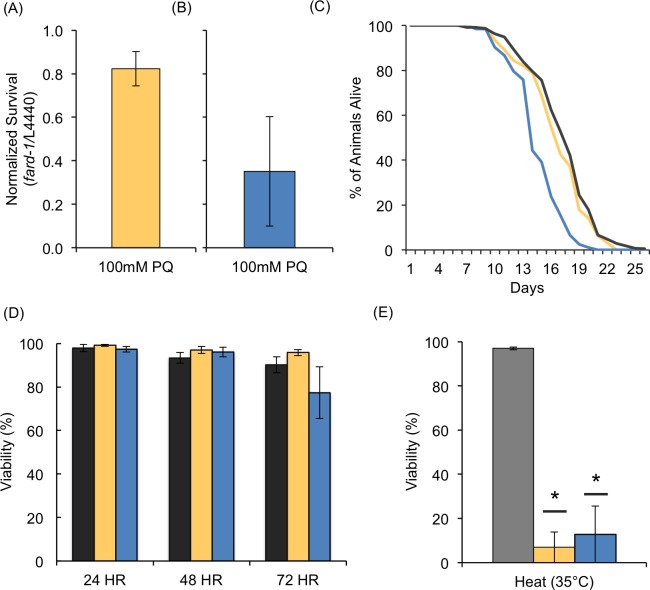
*fard-1* is Required for Thermotolerance but not Osmotic Stress in Adult Animals. (A) A sample of a large population of adult-only *fard-1* RNAi-treated animals was assessed for viability after 48 hours of 100mM PQ treatment in each population prepared for biochemical analysis. (B) Developmental *fard-1* RNAi-treated populations were assayed in 100mM PQ as well; however, fewer animals were needed as no HPLC-MS/MS was carried out on those animals. The data presented is normalized to the L4440 controls to account for the differences in culture size. Individual comparisons can be found as S5 Fig. (C) Lifespan analysis was completed for developmental *fard-1* (blue) and adult-only *fard-1* RNAi treatment (yellow). There was a significant reduction (p<0.001) in the average lifespan with developmental RNAi (13.4 ± 0.8 days) versus control (18.0 ±0.5 days). (D) Viability was assessed for the same *fard-1* treatments following exposure to 500mM NaCl and (E) after 24 hours of heat stress at 35°C. Data presented here is a result of at least 4 biological replicates containing a minimum of 40 animals per condition in each replicate. SEM is presented, and *p<0.05 was determined by unpaired t-tests using Holm-Sidak corrections for multiple comparisons where applicable.

The adult-only *fard-1* RNAi treatment is a useful tool to examine compromised ether lipid populations as developmental *fard-1* RNAi or loss-of-function mutation results in a near complete depletion of the ether lipid pools which is not reflective of many disease states. There is also significant remodeling of the PE population in developmental *fard-1* RNAi animals that alters the baseline lipid distribution and may influence how these membranes would respond to insults, when compared to the wild-type membrane. The adult-only *fard-1* RNAi treatment also affords an opportunity to examine the phenotypic consequences of perturbation of ether lipid synthesis depletion in a relatively uncompromised membrane. Unlike the developmental *fard-1* RNAi (Fig 6B) and the *fard-1* loss-of-function mutants [4], the adult-only *fard-1* treated animals are only slightly and not significantly sensitive to oxidative stress (Fig 6A). Additionally, adult-only RNAi did not impact longevity (Fig 6C). Taken together, survival in stress and control conditions is dependent on larval expression of *fard-1*. We hypothesize that its primary role may be to generate an initial membrane landscape that enables juvenile animals to respond to complex sets of environmental stressors, which is supported by the higher expression of ether lipid synthesis genes in larval animals.

There are other stress conditions that may require the synthesis of ether lipids in adult animals, and we examined the role of *fard-1* in heat stress and osmotic stress (Fig 6D and 6E). Although *fard-1* is not required for survival in elevated salt conditions, it is clear that adult-only *fard-1* plays a significant role in surviving elevated temperatures to the same extent as that was observed with developmental RNAi treatment (Fig 6E). Thus, the adult-only model will be of great utility in exploring how the phospholipid population and, in particular, the ether lipids respond to alternative insults.

## Discussion

Ether-linked phospholipids are unique membrane lipids that are prevalent in eukaryotes but whose biological function is not yet fully understood. We found that the abundance and structure of the ether lipid population in adult *C*. *elegans* is similar to that of humans. Specifically, the amount and distribution of ether lipids in the nematode very closely mirrors certain control cell lines used in the study of plasmalogen deficiency, with approximately 35% of phosphoethanolamine-containing lipids being P-PE, as compared to the nearly 30% seen in the adult nematode [19]. We utilized panels of standards to quantify the contribution of ether lipids in the nematode and found that approximately 10% of the major phospholipids contain an alkyl-ether or an alkenyl-ether bond. Although these predictions will be refined as more lipid standards become available, it is clear that ether-linkages are significantly represented in the nematode’s phospholipidome. Thus, our ether lipid profiles further corroborated and expanded the recent characterization of ether lipids in *C*. *elegans* [4] and increased the power of the nematode as a genetic and biochemical model to their continued study.

Through HPLC-based mass spectrometry, we are able not only to measure the overall abundance of ether-linkages in the major phospholipid classes but also to quantify the individual species. Our HPLC-MS/MS analysis compares intact phospholipid species and allowed for the consideration of overall properties of the molecule in addition to the distribution of individual fatty acid tails within the major lipid populations. This capability is valuable as the identity of the two fatty acid tails, their linkages, and the phospho-containing headgroup all contribute to the properties and the function of the molecule. In considering intact ether-linked phospholipids, we find far fewer major types of O-PE (8 species) and P-PE (7 species) present than the number of major unique species associated in the PE (21 species) and particularly in the PC (51 species) populations. Although the representation of ether-linked species is complete, the PE and PC lipids are considered by their combined chains and may be underrepresented [5]. This thorough mapping has allowed for the monitoring of changes in individual species and in the overall properties of the membrane upon ether lipid deficiency.

Using our HPLC-MS/MS generated profiles, we compared the types of phospholipids present in the nematode versus the phospholipids from characterized mammalian species. Although many of the features are similar, there is a marked absence of ether-linked species within phosphocholine-containing lipids of the nematode. The P-PC lipids found in mammals are often associated with cardiac tissue which may help to explain the absence in *C*. *elegans*; regardless, the lack of P-PC must be considered in the nematode model [2]. An additional difference in the plasmalogen population of *C*. *elegans* from that of mammals is the identity of the fatty acid located at the *sn-2* position. In humans, this position usually contains a polyunsaturated fatty acid, most commonly arachidonic acid (C20:4n6) or docosahexaenoic acid (C22:6n3) [2], while in *C*. *elegans*, the most common fatty acids are C18:1 and C18:2 as reported here and previously [4, 5]. One model for ether lipid function is that these molecules are a source of polyunsaturated fatty acids for phospholipase A2-mediated release; however, the absence of large amounts of polyunsaturated fat associated with ether-linked phospholipids argues against selective polyunsaturated fatty acid release as the primary function of these lipids in the nematode [2]. It is interesting to speculate that the ether lipids may serve as a unique repository for selective release of C18:1 and C18:2 fatty acids.

We were intrigued by the significant abundance of the O-PEs, which accumulate in a nearly 1:1 ratio with the P-PE population. The O-PE lipids make up more than 20% of the phosphoethanolamine lipids in adult nematodes, raising the possibility that they may serve an independent function. These O-PE lipids are present in mammals as well but little is known about their function except as P-PE precursors. Perhaps, these species may accumulate to maintain reserves for the rapid regeneration of the P-PE species when needed. We did see a greater reduction of O-PE than P-PE (40% versus 14%) after adult-only *fard-1* RNAi treatment, suggesting the preferential maintenance of the P-PE population over O-PE; however, the mechanism for this bias is not yet clear. The conversion of O-PE to P-PE is likely to be a regulated process that can be selectively induced; however, the plasmanylethanolamine desaturase responsible for O-PE to P-PE conversion has not yet been identified in *C*. *elegans*. The characterization of this enzyme would allow for the specific interrogation of O-PEs in the nematode [20].

In addition to examining the distribution of ether lipids in wild-type nematodes, we also compromised ether lipid synthesis *in vivo*. To do so, we targeted *fard-1*, which is required for generating the fatty alcohol needed for ether lipid production and is the proposed rate-limiting step of ether lipid synthesis [21]. When RNAi against *fard-1* is initiated in larval animals, there is near-total depletion of ether lipids and the plasmanylethanolamine precursors consistent with *fard-1* loss-of-function animals [4]. In contrast to mutant models, the application of RNAi for gene depletion can be initiated at different times in the life cycle of nematodes. Thus, we can separate the functions of ether-linked lipid synthesis during development versus in mature adults. This capability is particularly useful for the study of ether-linked lipids, as they are implicated in human disease in two distinct ways. First, compromised plasmalogen production due to inherited genetic mutations can directly result in RCDP and other Peroxisomal Biogenesis Disorders, and, consistent with this role, we find that ether-linked lipid synthesis in development plays a vital role in *C*. *elegans* stress sensitivity consistent with the stress phenotypes from *fard-1* mutants [4]. The specific roles of ether lipids in larvae are also consistent with the requirement for functional peroxisomes in nematode development, and further examination of plasmalogen populations in models of peroxisome deficiency will be informative [22, 23].

Second, altered ether lipid abundance in adults is correlated with many later-onset diseases including Alzheimer’s disease and many cancers [14, 16]. When *fard-1* RNAi is initiated specifically in adults, the treated nematodes have significant changes in their lipid profiles including reduced O-PE levels and severely compromised ether lipid production. However, the adult-only *fard-1* RNAi resulted in only a marginal sensitivity to oxidative stress, suggesting that overall abundance of ether lipids is more important to stress survival than new synthesis. This observation led us to consider the role of ether lipids in stress response as it has been suggested that these ether-linked species would be degraded under such conditions. Exposure to PQ in control and *fard-1* RNAi treated animals does not result in a general loss of ether lipids. Perhaps, specific membranes or domains would show a selective loss of the ether lipids that may act to protect specific membrane regions but which would be diluted by our analysis of the total phospholipid population. Detailed biochemical analysis of additional oxidative stress paradigms is also warranted, as there are multiple ways to impact the amount of oxidative stress *in vivo*. In the PQ studies presented here, there were no changes in the ether lipid profiles with two distinct PQ treatments. It is possible that the role of ether lipids is greater at different strengths or durations of stress exposure. An expanded study comparing stress sensitivity and lipid alterations would be useful to further understand this relationship as ether lipids likely function as only a part of the overall response to elevated stress.

The nature of the relationship between ether-linked lipids and stress was somewhat surprising to us. We had predicted that the introduction of oxidative stress would result in a depletion of these highly susceptible species; however, we did not see any reduction in O-PE or P-PE abundance with PQ exposure. Because of the toxicity of the PQ treatment, it was not possible to extend the exposure, as there would not be sufficient living animals remaining for analysis. We did test whether the increased ROS could induce ether lipid synthesis to compensate for the consumption of these lipids. In the adult-only *fard-1* RNAi treatment where ether lipid synthesis is severely compromised, there is no change in overall O-PE or P-PE abundance or the makeup of individual ether lipid species. The membrane lipids are not entirely unaffected by stress, indicating that not only is the PQ treatment effective but also that the membrane composition is, in fact, impacted by increased exposure to oxidative stress. Indeed, we have detected a specific loss of PC lipids, particularly those with highly susceptible C20 PUFA chains. Further work is required to determine if this reduction is a result of an active remodeling response or a loss from damage generated by ROS. Regardless, the reduction in PC lipids and not ether lipids is the primary effect of this PQ treatment in nematodes.

Because we did not observe consumption of ether lipids in our PQ studies, it will be important to consider other potential roles of ether lipids in stress that do not predict a significant depletion of the species. For instance, it is possible that a subset of ether lipids may act as a sensor to drive membrane remodeling in high stress conditions, and, in fact, lipid sensors that modulate composition through transcriptional activation have been identified [24]. Similarly, specific ether lipid species may function through other signaling pathways that would be beneficial to combating elevated levels of stress. Finally, the types of lipids found within the membrane have a significant impact on the properties of that membrane including its fluidity and permeability. Therefore, the role of ether lipids in stress response may be in the contribution of these species to the overall membrane landscape and function. This role would be supported by the greater stress sensitivity observed in developmental versus adult-only *fard-1* RNAi where there is a much more severe depletion of ether lipids. Additional work is needed to characterize the full impact of more localized changes in ether lipid populations as well as to explore other mechanisms that may drive the stress sensitivity in ether lipid deficiency. In particular, supplementation of ether lipids will be valuable in determining whether specific species mediate survival in stress or whether the general presence of an ether-linkage is sufficient for survival.

It is clear that developmental RNAi results in a greater impact on the health of the animal, and the major role of ether lipid biosynthesis may be in establishing proper membrane composition during development in the nematode. Not only was the expression of ether lipid biosynthesis genes greater in larval animals, but developmental *fard-1* RNAi treatment also resulted in greater stress sensitivity as well as a shortened lifespan. There is still active production of ether lipids in adult animals; however, it is unclear what role this low-level ether lipid synthesis may play. When ether lipid production is compromised in adults, there is not a corresponding decrease in the P-PE abundance at least in the short duration tested here. It is possible that ether lipids are more stable in adults, and turnover studies will help to determine the dynamics of these pools. Regardless, the synthesis of ether lipids in adults needs to be elucidated to increase the understanding of how their depletion may impact disease progression. We have found a significant role for *fard-1* in the adult animals after heat stress, suggesting that the role of ether lipids in thermotolerance may require active lipid synthesis and function through a different mechanism than oxidative stress response.

Using mass spectrometry, we have been able to map how the phospholipid populations change in animals lacking ether lipids and find a major shift in the overall membrane landscape by directly sampling the lipid populations with HPLC-based mass spectrometry. Additionally, we applied these biochemical tools to nematodes exposed to oxidative insults and can thoroughly map the alterations in membrane lipids after stress exposure. Ultimately, the nematode will be useful in understanding not only the role of ether-linked lipids in basic biology, but also their functions in membrane remodeling responses to environmental stressors.

## Material and Methods

### Nematode Strains and Growth Conditions

The temperature-sensitive sterile strain, CF512, *fer-15(b26);fem-1(hc17)*, was obtained from the *Caenorhabditis* Genetics Center (CGC, Minneapolis, MN) and maintained on *E*. *coli* (OP50) unless otherwise noted. CF512 animals were used for all experiments to allow for isolation of adults without larval contamination as previously described [5].

### RNAi Vectors and Feeding Protocols

HT115 bacteria, transformed with empty vector control RNAi (L4440) or *fard-1* RNAi from the Ahringer library, were grown in approximately 100mL LB (50μg/mL carbenicillin; 15μg/mL tetracycline) overnight. RNAi plates were prepared as previously described [5]. In short, bacteria were plated at a density of 0.15g per 10cm NGM-CI plate. L1 animals or young adult worms (44 hours from L1) were plated at a density of 2,500 worms per plate and incubated at 25°C for 48 hours.

### Gas Chromatography/Mass Spectrometry Analysis of Fatty Acid Chains

For fatty acid analysis, total lipids were extracted and major lipid groups (phospholipid and neutral lipid) were purified by solid phase exchange chromatography. The resulting lipids were resuspended in sulfuric methanol to generate fatty acid methyl esters as described [25]. FAMEs were analyzed by gas chromatography/mass spectrometry (GC/MS) (Agilent 5975GC, 6920MS).

### Extraction and Detection of Intact Phospholipids by HPLC-MS/MS

Total lipid fractions were isolated from frozen worm populations (5,000–10,000 animals) via chloroform:methanol (2:1) extraction [5]. Total lipid extracts were dried under nitrogen stream and then dissolved in 200–300μL of acetonitrile/2-propanol/water (65:30:5 v/v/v) dilution buffer [26]. As previously described, 10μL of each sample was injected onto a onto an HPLC system (Accela 600) equipped with a C18 Hypersil Gold 2.1 x 50mm, 1.9μm column (25002–052130; Thermo Scientific) and connected to an LTQ-Orbitrap mass spectrometer (Thermo Scientific) [5]. Analysis of the HPLC-MS/MS data was conducted using the software Lipid Data Analyzer (LDA) Version 1.6.2.5 [17]. The program utilizes exact mass, retention time, and predicted isotope distribution from full-profile, negative-ion-mode MS1 scans to identify lipids. The exact mass lists for PC, PE, O-PE and P-PE were provided to the program using the same molecular ranges previously described [5]. A 0.1% cutoff was applied in order to focus on the major phospholipid species in control animals, but, although it is only 0.08% abundant in control animals, we included the species P-18:0/20:2 in the final P-PE mass list, in order to compare the alkenyl form of the ether-linked lipid with its alkyl O-18:0/20:2 counterpart.

For phospholipid quantification, we employed a correction factor in order to correct for varying ionization efficiencies for the different lipid classes in our analysis. Because all species are not available, our correction factor was determined by the following representative species from Avanti Polar Lipids: 1,2-dilauroyl-sn-glycero-3-phosphoethanolamine (PE 12:0/12:0), 1,2-dilauroyl-sn-glycero-3-phosphocholine (PC 12:0/12:0), 1-(1Z-octadecenyl)-2-oleoyl-sn-glycero-3-phosphoethanolamine (P-PE 18:0/18:1), 1,2-dioleoyl-sn-glycero-3-phosphoethanolamine (PE 18:1/18:1), 1,2-dioleoyl-sn-glycero-3-phosphocholine (PC 18:1/18:1), and 1,2-dilignoceroyl-sn-glycero-3-phosphocholine (PC 20:4/20:4). When possible we included phospholipids with various fatty acids to account for differences in chain length and shape; however, we did not see any correlation with short versus long chains or different degrees of unsaturation. In summary, for each HPLC analysis, we used a correction factor 0.67580026 to account for the higher ionization efficiency of the PC’s over the PE population.

### *qRT-PCR* Analysis of Ether Lipid Synthesis Genes

Nematodes (~50μL pellet) were harvested after 0 hours (L1 stage), 44 hours (day 1 adult) and 92 hours (day 3 adults) on OP50. RNA was isolated using TRIzol (Invitrogen) and purified by RNeasy Mini Kit (Qiagen) and DNase I solution (Qiagen), and cDNA was made by ProtoScript First Strand cDNA Synthesis Kit (New England Biolabs). A 10μL reaction consisted of 5ng of cDNA, 1X Power SYBR Green PCR Master Mix (Life Technologies), and 5pmol each of the forward and reverse primers. The qRT-PCR reactions were executed on ABI 7900HT Fast Realtime PCR platforms and the cycle threshold (CT) values were exported from the Sequence Detection System 2.3 software. qRT-PCR efficiency values were calculated from mean CT values and were then used to calculate the relative expression of each gene as described [27], normalized to the expression of the housekeeping gene, *cdc-42*.

### Stress Sensitivity and Lifespan Analysis

To quantify the ability of the animals to survive in high oxidative stress conditions, 100–200 day 3 adult worms were exposed to 100mM paraquat (PQ) in M9 buffer. Samples were placed on horizontal shaker at room temperature, and survival percentage was determined in a blinded manner. Residual bacteria may have been present after transfer; however, bacteria was not added to the PQ treatment for either *fard-1* or L4440 RNAi conditions. Animal death was determined when worms did not respond after 2 gentle prods with a worm pick. HPLC-MS/MS analysis requires a minimum of 5,000 adult animals; therefore, larger-scale PQ treatment was performed at the same final concentration. From the HPLC-MS/MS studies, an aliquot was counted to obtain corresponding stress sensitivity numbers.

Lifespan assays were conducted as follows: L1s were plated onto OP50 and allowed to grow for 44 hours. The day 1 adults were then transferred to NGM-CI plates seeded with *fard-1* or L4440 RNAi bacteria. The worms were then counted each day and transferred to fresh plates every 2 to 3 days. To test for sensitivity to osmotic stress, day 3 animals (~50) were transferred to high salt plates (500mM NaCl) and scored every 24 hours for viability as described above. The NaCl plates were seeded with *fard-1* or L4440 control bacteria. Thermotolerance was determined by moving day 3 adults on seeded plates to a 35°C bath for 24 hours. Animals were allowed to recover for 24 hours at 25°C before assayed for viability.

### ^15^N-Incorporation Assays

^15^N feeding plates were prepared as previously described [5]. Briefly, LB (^14^N) and ^15^N–Isogro (Sigma) cultures were inoculated with OP50 and grown overnight. Once harvested, the 0.15g of bacteria were plated on individual agarose plates. Approximately 5,000 synchronized worms were harvested after 18 hours on isotope feeding plates, and lipids were extracted for analysis on HPLC-MS/MS. ^15^N-isotope incorporation rates were determined as previously described using the Thermo Qual Browser Software in Xcalibur version 2.2 (Thermo Scientific). New phospholipid synthesis was calculated by the following formula: [(Σm+1,…,m+4)/(Σm+0,…,m+4)]*100 as reported in [5].

## Supporting Information

S1 Fig The Majority of Progeny Production Occurs By Day 3 of Adulthood. In order to demonstrate that our analysis is reflective of adult metabolism, we assayed the production of progeny at 25°C under our laboratory conditions in control L4440 (black) and *fard-1* RNAi treated (blue) animals. The lipid analysis described was conducted at Day 3 when the majority (>95%) of progeny production is completed. Brood analysis was performed on at least 8 individual animals, and SEM is shown.
(DOCX)

S2 Fig GC-MS Analysis of Fatty Acid Tails After Developmental *fard-1* RNAi. Developmental *fard-1* RNAi-treatment results in a significant re-distribution of fatty acid tails in both neutral lipid (A) and phospholipid (B) populations as assessed by GC-MS (Dancy et al, 2015). The altered fatty acid abundance is significant only in fatty acids containing 18 carbons which are represented above. Data shown are from at least 3 experimental replicates, SEM is shown. *p<0.05 was determined by unpaired t-tests using Holm-Sidak corrections for multiple comparisons.
(DOCX)

S3 Fig Longer Adult-Only *fard-1* RNAi Further Alters Fatty Acid Composition. The changes in the overall fatty acid composition upon 2 days of adult-only *fard-1* RNAi (light blue) were compared to animals fed adult-only *fard-1* RNAi for 7 days (dark blue). To control for the changes in fatty acid content that occur over aging, we normalized to age-matched L4440 RNAi controls. The elevated C18:0 abundance is indicative of decreased ether-lipid abundance; however, this increase is only significant at a P-Value of 0.093 (#). There is not a corresponding change in the amount of C18:1n7, a common feature of *fard-1* RNAi, which warrants further investigation. Data is from three biological replicates with SEM is shown.
(DOCX)

S4 Fig Two Distinct Paraquat Treatments Result in Indistinguishable Lipid Alterations. In addition to exposure to 100mM PQ for 2 days (dark red), nematodes were subjected to a longer, 4 day PQ treatment (light red). Because 100mM PQ is toxic to wild-type nematodes after 4 days, a lower dose of PQ (25mM) was used. When normalized to age-matched controls, there were no significant differences as assessed by unpaired t-tests in total abundance of ether lipids (A), the distribution of ether-linked lipids (B) or the remodeling of the PC and PE populations following PQ exposure (C). Data shown are from at least 3 experimental replicates, SEM is shown.
(DOCX)

S5 Fig Developmental RNAi of *fard-1* Results in Significant Stress Sensitivity. (A) There was significant variability in survival under PQ treatment; however, it is clear that developmental *fard-1* RNAi resulted in significant reduction in survivorship when subjected to 100mM PQ in M9 buffer for 72 hours. This treatment was done on a small scale to confirm the oxidative stress phenotype of *fard-1* loss-of-function animals (Shi et al, 2016). (B) Adult-only *fard-1* RNAi treatment resulted in a reduced survivorship in 100mM PQ, but this change was not statistically significant (p = 0.15). These animals were counted at 48 hours, and the experiments were performed on a large-scale in order to process the same animals for lipid analysis. Data shown are from 5 biological replicates for developmental RNAi and from 12 replicates for adult-only RNAi. SEM is shown, and *p<0.05 was determined by unpaired t-tests.
(DOCX)

S1 Dataset Phospholipid Abundance in Control Versus Developmental *fard-1-*RNAi Treated Animals. (XLSX)

S2 Dataset Phospholipid Abundance in Adult-Only *fard-1* RNAi-Treated Animals. (XLSX)

S3 Dataset Phospholipid Abundance After Paraquat Treatment in Control and Adult-Only *fard-1* Treatment. (XLSX)

## References

[1] Nagan N, Zoeller RA. Plasmalogens: biosynthesis and functions. Progress in lipid research. 2001;40(3):199–229. Epub 2001/03/29. 11275267 10.1016/s0163-7827(01)00003-0

[2] Braverman NE, Moser AB. Functions of plasmalogen lipids in health and disease. Biochim Biophys Acta. 2012;1822(9):1442–52. 10.1016/j.bbadis.2012.05.008 22627108

[3] Tanaka T, Ikita K, Ashida T, Motoyama Y, Yamaguchi Y, Satouchi K. Effects of growth temperature on the fatty acid composition of the free-living nematode Caenorhabditis elegans. Lipids. 1996;31(11):1173–8. Epub 1996/11/01. 8934450 10.1007/BF02524292

[4] Shi X, Tarazona P, Brock TJ, Browse J, Feussner I, Watts JL. A Caenorhabditis elegans model for ether lipid biosynthesis and function. Journal of lipid research. 2016;57(2):265–75. Epub 2015/12/20. PubMed Central PMCID: PMCPmc4727422. 10.1194/jlr.M064808 26685325 PMC4727422

[5] Dancy BC, Chen SW, Drechsler R, Gafken PR, Olsen CP. 13C- and 15N-Labeling Strategies Combined with Mass Spectrometry Comprehensively Quantify Phospholipid Dynamics in C. elegans. PLoS One. 2015;10(11):e0141850 Epub 2015/11/04. PubMed Central PMCID: PMCPmc4631354. 10.1371/journal.pone.0141850 26528916 PMC4631354

[6] Zoeller RA, Morand OH, Raetz CR. A possible role for plasmalogens in protecting animal cells against photosensitized killing. The Journal of biological chemistry. 1988;263(23):11590–6. Epub 1988/08/15. 3403547

[7] Bochkov VN, Oskolkova OV, Birukov KG, Levonen AL, Binder CJ, Stockl J. Generation and biological activities of oxidized phospholipids. Antioxidants & redox signaling. 2010;12(8):1009–59. Epub 2009/08/19. PubMed Central PMCID: PMCPmc3121779. 19686040 10.1089/ars.2009.2597 PMC3121779

[8] Aldini G, Dalle-Donne I, Colombo R, Maffei Facino R, Milzani A, Carini M. Lipoxidation-derived reactive carbonyl species as potential drug targets in preventing protein carbonylation and related cellular dysfunction. ChemMedChem. 2006;1(10):1045–58. Epub 2006/08/18. 10.1002/cmdc.200600075 16915603

[9] Broniec A, Klosinski R, Pawlak A, Wrona-Krol M, Thompson D, Sarna T. Interactions of plasmalogens and their diacyl analogs with singlet oxygen in selected model systems. Free radical biology & medicine. 2011;50(7):892–8. Epub 2011/01/18. PubMed Central PMCID: PMCPmc3073128. 21236336 10.1016/j.freeradbiomed.2011.01.002 PMC3073128

[10] Stadelmann-Ingrand S, Favreliere S, Fauconneau B, Mauco G, Tallineau C. Plasmalogen degradation by oxidative stress: production and disappearance of specific fatty aldehydes and fatty alpha-hydroxyaldehydes. Free radical biology & medicine. 2001;31(10):1263–71. Epub 2001/11/14. 11705705 10.1016/s0891-5849(01)00720-1

[11] Brites P, Ferreira AS, da Silva TF, Sousa VF, Malheiro AR, Duran M, et al Alkyl-glycerol rescues plasmalogen levels and pathology of ether-phospholipid deficient mice. PLoS One. 2011;6(12):e28539 PubMed Central PMCID: PMCPMC3232224. 10.1371/journal.pone.0028539 22163031 PMC3232224

[12] Wanders RJ. Peroxisomes in human health and disease: metabolic pathways, metabolite transport, interplay with other organelles and signal transduction. Sub-cellular biochemistry. 2013;69:23–44. Epub 2013/07/04. 10.1007/978-94-007-6889-5_2 23821141

[13] Kaddurah-Daouk R, McEvoy J, Baillie R, Zhu H, J KY, Nimgaonkar VL, et al Impaired plasmalogens in patients with schizophrenia. Psychiatry research. 2012;198(3):347–52. Epub 2012/04/20. 10.1016/j.psychres.2012.02.019 22513041

[14] Kou J, Kovacs GG, Hoftberger R, Kulik W, Brodde A, Forss-Petter S, et al Peroxisomal alterations in Alzheimer's disease. Acta neuropathologica. 2011;122(3):271–83. Epub 2011/05/20. PubMed Central PMCID: PMCPmc3168371. 10.1007/s00401-011-0836-9 21594711 PMC3168371

[15] Donovan EL, Pettine SM, Hickey MS, Hamilton KL, Miller BF. Lipidomic analysis of human plasma reveals ether-linked lipids that are elevated in morbidly obese humans compared to lean. Diabetology & metabolic syndrome. 2013;5(1):24. Epub 2013/05/16. PubMed Central PMCID: PMCPmc3663699. 23672807 10.1186/1758-5996-5-24 PMC3663699

[16] Benjamin DI, Cozzo A, Ji X, Roberts LS, Louie SM, Mulvihill MM, et al Ether lipid generating enzyme AGPS alters the balance of structural and signaling lipids to fuel cancer pathogenicity. Proceedings of the National Academy of Sciences of the United States of America. 2013;110(37):14912–7. Epub 2013/08/28. PubMed Central PMCID: PMCPmc3773741. 10.1073/pnas.1310894110 23980144 PMC3773741

[17] Hartler J, Trotzmuller M, Chitraju C, Spener F, Kofeler HC, Thallinger GG. Lipid Data Analyzer: unattended identification and quantitation of lipids in LC-MS data. Bioinformatics (Oxford, England). 2011;27(4):572–7. Epub 2010/12/21. 10.1093/bioinformatics/btq699 21169379

[18] Dorninger F, Brodde A, Braverman NE, Moser AB, Just WW, Forss-Petter S, et al Homeostasis of phospholipids—The level of phosphatidylethanolamine tightly adapts to changes in ethanolamine plasmalogens. Biochim Biophys Acta. 2015;1851(2):117–28. Epub 2014/12/03. PubMed Central PMCID: PMCPmc4331674. 10.1016/j.bbalip.2014.11.005 25463479 PMC4331674

[19] Gaposchkin DP, Farber HW, Zoeller RA. On the importance of plasmalogen status in stimulated arachidonic acid release in the macrophage cell line RAW 264.7. Biochim Biophys Acta. 2008;1781(4):213–9. Epub 2008/03/11. 10.1016/j.bbalip.2008.01.007 18328831

[20] Watschinger K, Werner ER. Orphan enzymes in ether lipid metabolism. Biochimie. 2013;95(1):59–65. Epub 2012/07/10. PubMed Central PMCID: PMCPmc3520006. 10.1016/j.biochi.2012.06.027 22771767 PMC3520006

[21] Honsho M, Asaoku S, Fujiki Y. Posttranslational regulation of fatty acyl-CoA reductase 1, Far1, controls ether glycerophospholipid synthesis. The Journal of biological chemistry. 2010;285(12):8537–42. Epub 2010/01/15. PubMed Central PMCID: PMCPmc2838275. 10.1074/jbc.M109.083311 20071337 PMC2838275

[22] Simmer F, Moorman C, van der Linden AM, Kuijk E, van den Berghe PV, Kamath RS, et al Genome-wide RNAi of C. elegans using the hypersensitive rrf-3 strain reveals novel gene functions. PLoS biology. 2003;1(1):E12 Epub 2003/10/14. PubMed Central PMCID: PMCPmc212692. 10.1371/journal.pbio.0000012 14551910 PMC212692

[23] Sonnichsen B, Koski LB, Walsh A, Marschall P, Neumann B, Brehm M, et al Full-genome RNAi profiling of early embryogenesis in Caenorhabditis elegans. Nature. 2005;434(7032):462–9. Epub 2005/03/26. 10.1038/nature03353 15791247

[24] Holthuis JC, Menon AK. Lipid landscapes and pipelines in membrane homeostasis. Nature. 2014;510(7503):48–57. Epub 2014/06/06. 10.1038/nature13474 24899304

[25] Perez CL, Van Gilst MR. A 13C isotope labeling strategy reveals the influence of insulin signaling on lipogenesis in C. elegans. Cell metabolism. 2008;8(3):266–74. Epub 2008/09/03. 10.1016/j.cmet.2008.08.007 18762027

[26] Bird SS, Marur VR, Sniatynski MJ, Greenberg HK, Kristal BS. Serum lipidomics profiling using LC-MS and high-energy collisional dissociation fragmentation: focus on triglyceride detection and characterization. Analytical chemistry. 2011;83(17):6648–57. Epub 2011/07/22. PubMed Central PMCID: PMCPmc3165109. 10.1021/ac201195d 21774539 PMC3165109

[27] Pfaffl MW. A new mathematical model for relative quantification in real-time RT-PCR. Nucleic acids research. 2001;29(9):e45 Epub 2001/05/09. PubMed Central PMCID: PMCPmc55695. 11328886 10.1093/nar/29.9.e45 PMC55695

