# Supplementary material for: HPLC-Based Mass Spectrometry Characterizes the Phospholipid Alterations in Ether-Linked Lipid Deficiency Models Following Oxidative Stress
Source: PLoS One. 2016 Nov 28;11(11):e0167229. doi: 10.1371/journal.pone.0167229 (PMC5125691; doi:10.1371/journal.pone.0167229)
Supplement: S1 Fig — In order to demonstrate that our analysis is reflective of adult metabolism, we assayed the production of progeny at 25°C under our laboratory conditions in control L4440 (black) and fard-1 RNAi treated (blue) animals. The lipid analysis described was conducted at Day 3 when the majority (>95%) of progeny production is completed. Brood analysis was performed on at least 8 individual animals, and SEM is shown. (DOCX) [file pone.0167229.s001.docx]

**S1 Fig. The Majority of Progeny Production Occurs By Day 3 of Adulthood.**

In order to demonstrate that our analysis is reflective of adult metabolism, we assayed the production of progeny at 25 °C under our laboratory conditions in control *L4440* (black) and *fard-1* RNAi treated (blue) animals. The lipid analysis described was conducted at Day 3 when the majority (>95%) of progeny production is completed. Brood analysis was performed on at least 8 individual animals, and SEM is shown.
