# Supplementary material for: HPLC-Based Mass Spectrometry Characterizes the Phospholipid Alterations in Ether-Linked Lipid Deficiency Models Following Oxidative Stress
Source: PLoS One. 2016 Nov 28;11(11):e0167229. doi: 10.1371/journal.pone.0167229 (PMC5125691; doi:10.1371/journal.pone.0167229)
Supplement: S2 Fig — Developmental fard-1 RNAi-treatment results in a significant re-distribution of fatty acid tails in both neutral lipid (A) and phospholipid (B) populations as assessed by GC-MS (Dancy et al, 2015). The altered fatty acid abundance is significant only in fatty acids containing 18 carbons which are represented above. Data shown are from at least 3 experimental replicates, SEM is shown. *p<0.05 was determined by unpaired t-tests using Holm-Sidak corrections for multiple comparisons. (DOCX) [file pone.0167229.s002.docx]

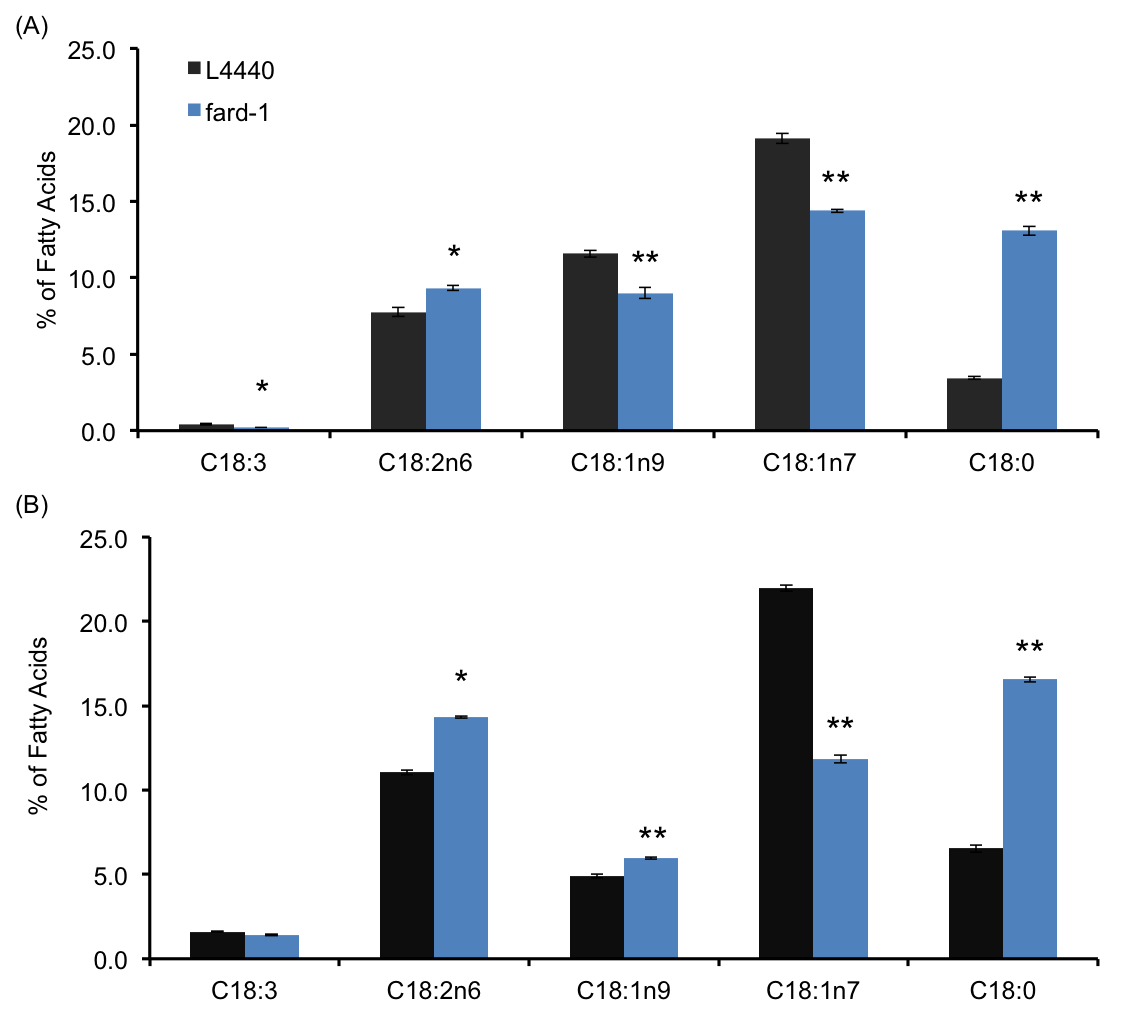


**S2 Fig. GC-MS Analysis of Fatty Acid Tails After Developmental *fard-1* RNAi.**

Developmental *fard-1* RNAi-treatment results in a significant re-distribution of fatty acid tails in both neutral lipid (A) and phospholipid (B) populations as assessed by GC-MS (Dancy et al, 2015). The altered fatty acid abundance is significant only in fatty acids containing 18 carbons which are represented above. Data shown are from at least 3 experimental replicates, SEM is shown. *p<0.05 was determined by unpaired t-tests using Holm-Sidak corrections for multiple comparisons.
