# Supplementary material for: HPLC-Based Mass Spectrometry Characterizes the Phospholipid Alterations in Ether-Linked Lipid Deficiency Models Following Oxidative Stress
Source: PLoS One. 2016 Nov 28;11(11):e0167229. doi: 10.1371/journal.pone.0167229 (PMC5125691; doi:10.1371/journal.pone.0167229)
Supplement: S3 Fig — The changes in the overall fatty acid composition upon 2 days of adult-only fard-1 RNAi (light blue) were compared to animals fed adult-only fard-1 RNAi for 7 days (dark blue). To control for the changes in fatty acid content that occur over aging, we normalized to age-matched L4440 RNAi controls. The elevated C18:0 abundance is indicative of decreased ether-lipid abundance; however, this increase is only significant at a P-Value of 0.093 (#). There is not a corresponding change in the amount of C18:1n7, a common feature of fard-1 RNAi, which warrants further investigation. Data is from three biological replicates with SEM is shown. (DOCX) [file pone.0167229.s003.docx]

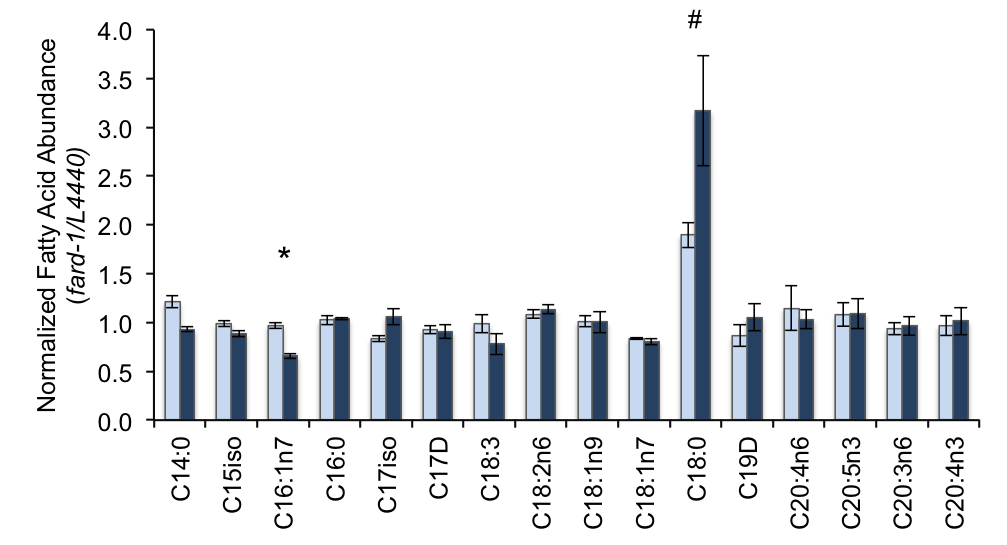


**S3 Fig. Longer Adult-Only *fard-1* RNAi Further Alters Fatty Acid Composition.**

The changes in the overall fatty acid composition upon 2 days of adult-only *fard-1* RNAi (light blue) were compared to animals fed adult-only *fard-1* RNAi for 7 days (dark blue). To control for the changes in fatty acid content that occur over aging, we normalized to age-matched *L4440* RNAi controls. The elevated C18:0 abundance is indicative of decreased ether-lipid abundance; however, this increase is only significant at a P-Value of 0.093 (#). There is not a corresponding change in the amount of C18:1n7, a common feature of *fard-1* RNAi, which warrants further investigation. Data is from three biological replicates with SEM is shown.
