# Supplementary material for: HPLC-Based Mass Spectrometry Characterizes the Phospholipid Alterations in Ether-Linked Lipid Deficiency Models Following Oxidative Stress
Source: PLoS One. 2016 Nov 28;11(11):e0167229. doi: 10.1371/journal.pone.0167229 (PMC5125691; doi:10.1371/journal.pone.0167229)
Supplement: S4 Fig — In addition to exposure to 100mM PQ for 2 days (dark red), nematodes were subjected to a longer, 4 day PQ treatment (light red). Because 100mM PQ is toxic to wild-type nematodes after 4 days, a lower dose of PQ (25mM) was used. When normalized to age-matched controls, there were no significant differences as assessed by unpaired t-tests in total abundance of ether lipids (A), the distribution of ether-linked lipids (B) or the remodeling of the PC and PE populations following PQ exposure (C). Data shown are from at least 3 experimental replicates, SEM is shown. (DOCX) [file pone.0167229.s004.docx]

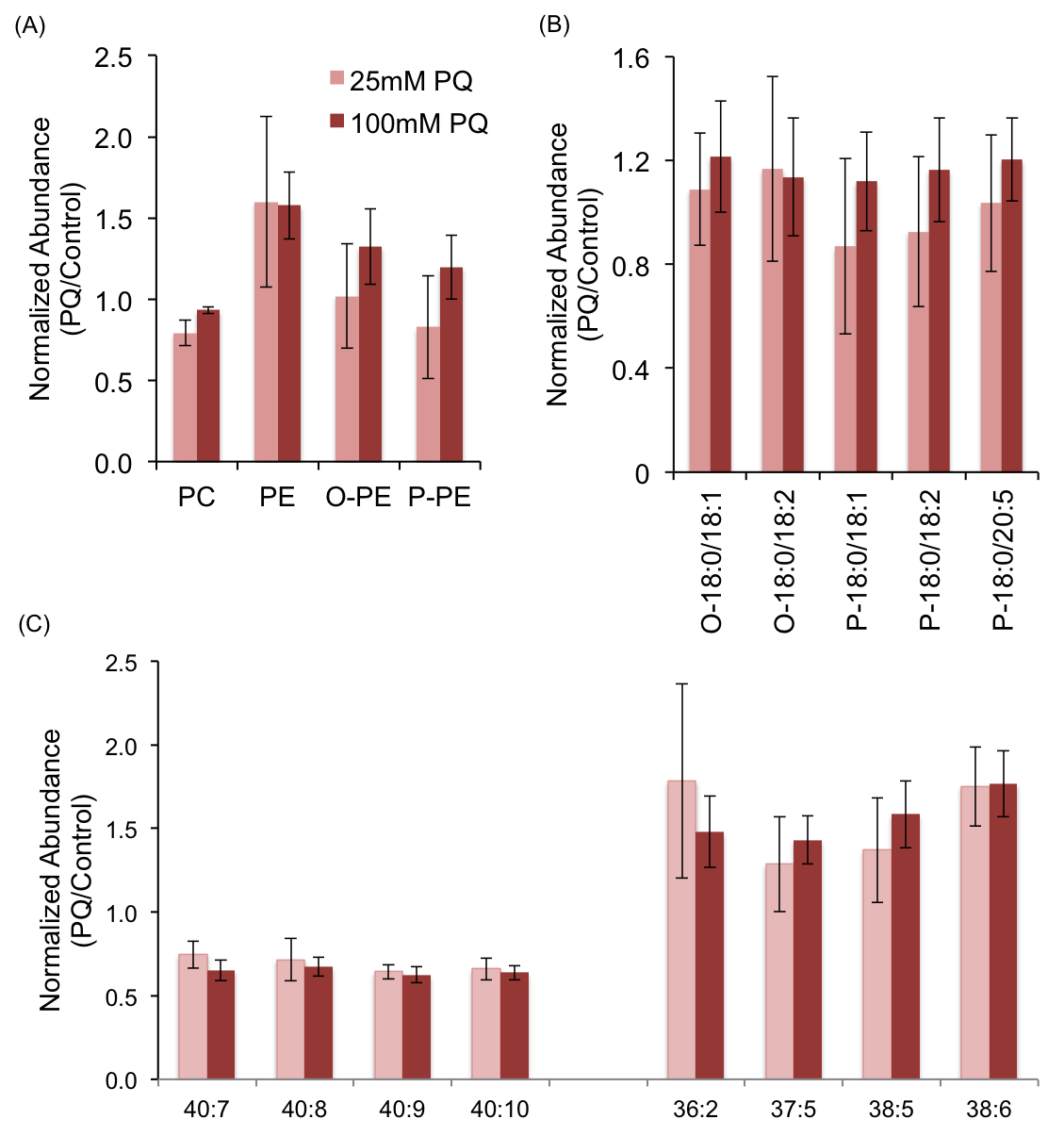


**S4 Fig. Two Distinct Paraquat Treatments Result in Indistinguishable Lipid Alterations.**

In addition to exposure to 100mM PQ for 2 days (dark red), nematodes were subjected to a longer, 4 day PQ treatment (light red). Because 100mM PQ is toxic to wildtype nematodes after 4 days, a lower dose of PQ (25mM) was used. When normalized to age-matched controls, there were no significant differences as assessed by unpaired t-tests in total abundance of ether lipids (A), the distribution of ether-linked lipids (B) or the remodeling of the PC and PE populations following PQ (C). Data shown are from at least 3 experimental replicates, SEM is shown.
