# Supplementary material for: HPLC-Based Mass Spectrometry Characterizes the Phospholipid Alterations in Ether-Linked Lipid Deficiency Models Following Oxidative Stress
Source: PLoS One. 2016 Nov 28;11(11):e0167229. doi: 10.1371/journal.pone.0167229 (PMC5125691; doi:10.1371/journal.pone.0167229)
Supplement: S5 Fig — (A) There was significant variability in survival under PQ treatment; however, it is clear that developmental fard-1 RNAi resulted in significant reduction in survivorship when subjected to 100mM PQ in M9 buffer for 72 hours. This treatment was done on a small scale to confirm the oxidative stress phenotype of fard-1 loss-of-function animals (Shi et al, 2016). (B) Adult-only fard-1 RNAi treatment resulted in a reduced survivorship in 100mM PQ, but this change was not statistically significant (p = 0.15). These animals were counted at 48 hours, and the experiments were performed on a large-scale in order to process the same animals for lipid analysis. Data shown are from 5 biological replicates for developmental RNAi and from 12 replicates for adult-only RNAi. SEM is shown, and *p<0.05 was determined by unpaired t-tests. (DOCX) [file pone.0167229.s005.docx]

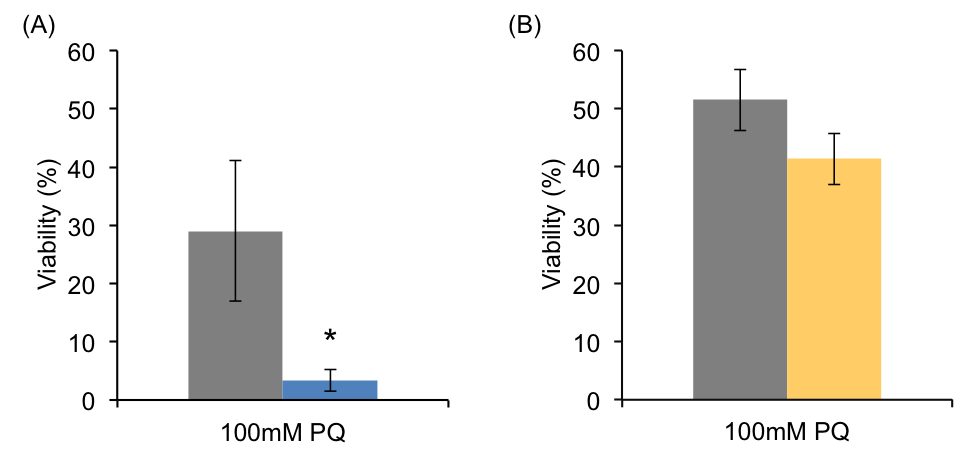


**S5 Fig. Developmental RNAi of *fard-1* Results in Significant Stress Sensitivity.**

(A) There was significant variability in survival under PQ treatment; however, it is clear that developmental *fard-1* RNAi resulted in significant reduction in survivorship when subjected to 100mM PQ in M9 buffer after 72 hours. This treatment was done in small scale to confirm the oxidative stress phenotype of *fard-1* loss-of-function animals (Shi et al, 2016). (B) Adult-only *fard-1* RNAi resulted in a reduced survivorship in 100mM PQ, but this change was not statistically significant (p=0.15). These animals were counted at 48 hours and performed in large-scale in order to process the same animals for lipid analysis. Data shown are from 5 biological replicates for developmental RNAi and from 12 replicates for adult-only RNAi. SEM is shown, and *p<0.05 was determined by unpaired t-tests.
